# A Sensitive Thresholding Method for Confocal Laser Scanning Microscope Image Stacks of Microbial Biofilms

**DOI:** 10.1038/s41598-018-31012-5

**Published:** 2018-08-29

**Authors:** Ting L. Luo, Marisa C. Eisenberg, Michael A. L. Hayashi, Carlos Gonzalez-Cabezas, Betsy Foxman, Carl F. Marrs, Alexander H. Rickard

**Affiliations:** 1 Department of Epidemiology, University of Michigan School of Public Health, Ann Arbor, MI USA; 2 Department of Cariology, Restorative Sciences and Endodontics, University of Michigan School of Dentistry, Ann Arbor, MI USA

## Abstract

Biofilms are surface-attached microbial communities whose architecture can be captured with confocal microscopy. Manual or automatic thresholding of acquired images is often needed to help distinguish biofilm biomass from background noise. However, manual thresholding is subjective and current automatic thresholding methods can lead to loss of meaningful data. Here, we describe an automatic thresholding method designed for confocal fluorescent signal, termed the biovolume elasticity method (BEM). We evaluated BEM using confocal image stacks of oral biofilms grown in pooled human saliva. Image stacks were thresholded manually and automatically with three different methods; Otsu, iterative selection (IS), and BEM. Effects on biovolume, surface area, and number of objects detected indicated that the BEM was the least aggressive at removing signal, and provided the greatest visual and quantitative acuity of single cells. Thus, thresholding with BEM offers a sensitive, automatic, and tunable method to maintain biofilm architectural properties for subsequent analysis.

## Introduction

Biofilms are architecturally ornate surface-attached microbial communities that exist throughout nature^1^. The biological activities of biofilms vary by ecological niche^2,3^ and particular attention has focused on the ability of biofilms to have deleterious effects^4,5^. For example, in humans, biofilms can cause chronic wounds and a multitude of diseases^6–8^. In industry and infrastructure, uncontrolled biofilm growth on ship hulls or on piping can interfere with function^9–11^. Much of the biological activity of biofilms is attributable to community composition and biofilm architecture.

To quantify biofilm architecture, a three-dimensional dataset is preferably generated from biofilms with intact structural integrity. One tool that provides the ability to generate three-dimensional datasets is a confocal laser scanning microscope (CLSM), which can capture two-dimensional cross-sections of a biofilm to produce a three-dimensional representation^12^. Cross-sections that make-up a confocal stack data often consist of 8-bit grayscale values from 0–255, which correspond to the intensity of signal captured, but images with higher bit depths can be generated (e.g. 12 bit, which corresponds to gray scale values from 0–4095). Following thresholding, the digital data contained within confocal stacks can be quantified by image analysis software such as COMSTAT^13^, Icy^14^, and PHLIP^15^, or imported to MatLab (Mathworks, Natick, MA) for customized analysis^16,17^.

Thresholding classifies pixels of a grayscale image as foreground biomass or background interstitial space using a cut-off value that represents the pixel’s signal brightness/intensity^18–20^. Thresholding can be performed on a two-dimensional image or a three-dimensional confocal stack^21,22^. A threshold that is too low will lead to false positives that will infer spatial presence of biomass when there is none. Conversely, a threshold that is too high will lead to false negatives: missing measurement of true biomass emitting low-intensity signal. In either case, suboptimal thresholding will bias measured features of the biofilm architecture. Thresholding can be done manually or automatically. Manual thresholding relies on individual(s), often operating under guidelines, for visually determining thresholds. This method can be arbitrary and the reproducibility/generalizability of results can be affected by inter-operator subjectivity^16,23,24^. By contrast, automatic thresholding eliminates subjectivity in thresholding; however, algorithm selection can drive sensitivity/specificity of regions of interest detection and predicate the success or failure of downstream outcome measurement^25^. Further, imaging platforms can affect algorithm performance (e.g. CLSM vs light microscopy) as can within-platform acquisition parameters (e.g. gain, smart offset, and excitation energy in CLSM)^21^. In the absence of a consensus on the best algorithm to automatically threshold CLSM images, manual thresholding has been used^26,27^.

Two algorithms used for automatically thresholding biofilm images that have been captured with confocal laser scanning microscopy are Otsu and the iterative selection (IS) methods^28,29^. Otsu’s method (which is included in the COMSTAT package) selects a threshold that maximizes between-class (background vs. foreground) variance^28^. Thus, this method is particularly powerful for separating foreground signal from background noise in images characterized with a bimodal intensity histogram^21,30^. The IS method has demonstrated the most congruency with manually-set thresholds for light and confocal biofilm images^30^. This method seeks to find a threshold that maximizes the separation between mean background and foreground values^29^. Functionally, Otsu and IS are similar and assume that histograms of image intensity values possess similarly-sized bimodal peaks that resemble a normal distribution^31,32^. CLSM images, however, are often characterized by unimodal histograms with long tails^33^. These characteristics are poorly compatible with implicit assumptions of IS/Otsu’s methods and are not well-matched for its use with confocal images^32,34^. In the case of CLSM images with long tails, IS/Otsu sacrifice actual biofilm material in favor of maximizing the separation between apparent foreground and background. This limitation led us to develop an automatic thresholding method designed to cope with unique features of CLSM image histograms, which we call the biovolume elasticity method (BEM).

## Results

### Consistency and agreement within and between automatic and manual thresholding

To evaluate the biovolume elasticity method (BEM) compared to other thresholding methods, we used images of oral biofilms treated and not treated (control) with water (Table 1). The biovolume elasticity method (BEM) thresholds for each image were lower than the thresholds calculated by IS and Otsu’s methods or those set manually. Additionally, the BEM had the least variance of all the methods. Overall, manual thresholding was consistently more aggressive at removing signal than BEM, but less aggressive than IS and Otsu. Each method produced slightly higher average thresholds for images of biofilms that had been treated intermittently with water. The difference in average thresholds between water treated and control group images was not statistically significant for all four methods.

**Table 1:** Average Biofilm Outcomes by Thresholding Method, Stratified by Treatment.

Outcomes by Method	Control Average (Standard Deviation)	Treatment Average (Standard Deviation)	Effect Size (p-value)^a^
BEM Threshold	11.360 (1.411)	11.96 (1.060)	0.234 (0.096)
Otsu’s Method Threshold	64.520 (13.257)	65.280 (10.550)	0.032 (0.824)
IS Threshold	66.400 (13.200)	67.280 (10.450)	0.037 (0.795)
Manual Threshold^b^	28.688 (6.279)	30.016 (4.531)	0.120 (0.396)
BEM Biovolume^c^	2,725,836 (1,570,653)	1,771,797 (634,596)	0.370 (**0.004**)
Otsu Biovolume	1,033,577 (606,285)	616,872 (261,495)	0.408 (**0.002**)
IS Biovolume	1,007,618 (594,215)	599,956 (259,985)	0.406 (**0.002**)
Manual^b^ Biovolume	1,811,782 (1,061,797)	1,091,577 (394,597)	0.410 (**0.002**)
BEM Surface Area^d^	2,416,820 (1,086,278)	1,983,686 (532,132)	0.245 (**0.041**)
Otsu Surface Area	1,261,839 (617,260)	877,151 (214,299)	0.384 (**0.003**)
IS Surface Area	1,246,216 (615,090)	861,151 (212,960)	0.386 (**0.003**)
Manual Surface Area	1,660,971 (761,391)	1,273,571 (310,737)	0.316 (**0.012**)
BEM Objects	3,526 (2,066)	2,521 (1,029)	0.294 (**0.018**)
Otsu Objects	2,018 (991)	2,379 (1,091)	0.171 (0.114)
IS Objects	2,051 (991)	2,433 (1,094)	0.180 (0.101)
Manual Objects	1,370 (814)	1,244 (634)	0.086 (0.272)

Fifty CLSM image stacks of oral biofilms were thresholded using four different methods and post-threshold biovolume, surface area, and number of objects were calculated. Half the biofilms imaged had been treated with water 8 and 18 hours into their 22 hour development and were designated as treatment biofilms. The other half were developed undisturbed over 22 hours and designated as control biofilms. Otsu and IS thresholds were significantly higher than BEM and manual thresholds and with higher standard deviation. BEM thresholds had the lowest standard deviation. Measured biovolume, surface area, and objects detected were highest for BEM, followed by manual, Otsu, and IS. Significance in the number of objects detected between treatment and control was detected with BEM and manual thresholds and not detected with Otsu/IS thresholds. Treatment reduced biovolume and surface area in all four methods. Outcomes varied by up to five-fold depending on threshold as in the case of objects detected in control images. Effect size between control and treatment groups was calculated with Cohen’s D, which quantifies the standardized difference of two means.

^a^Test performed was a 2-tailed student’s t-test for thresholds and 1-tailed student’s t-test for biofilm architectural outcomes. ^b^Manual threshold used for an image is the average value from five different operators for that image, rounded to the nearest whole number. ^c^Biovolume measured by count of total voxels post-thresholding. ^d^Surface area measured by count of total exposed surfaces post-thresholding.

Differences in magnitude between each pairwise combination of thresholding methods were evaluated with student’s paired t-test (summarized in Table 2). Regardless of treatment status, each pairwise comparison of methods was statistically significant, indicating differences between any two thresholding methods. Although Otsu and IS mean thresholds were close to each other, IS thresholds consistently scored two intensity units higher than Otsu thresholds on the same images, which minimized variance. Considering downstream quantification and rendering outcomes, the significance of a two unit difference in threshold is negligible.

**Table 2:** Comparison of Average Thresholds by Each Thresholding Method, Stratified by Treatment.

Pairwise Thresholding Method Comparison	Control Images (n = 25)			Treatment Images (n = 25)		
	Mean Threshold	Group 1 - Group 2 (95% confidence interval)	Paired t-test p-value	Mean Threshold	Group 1 - Group 2 (95% confidence interval)	Paired t-test p-value
Manual avg^a^. vs BEM	28.69/11.36	17.33 (14.47, 20.19)	<0.01	30.02/11.96	18.06 (16.09, 20.02)	<0.01
Manual avg. vs Otsu	28.69/64.52	−35.82 (−39.44, −32.22)	<0.01	30.02/65.28	−35.26 (−38.48, −32.04)	<0.01
Manual avg. vs IS	28.69/66.40	−37.71 (−41.30, −34.13)	<0.01	30.02/67.28	−38.26 (−40.45, −34.08)	<0.01
BEM vs Otsu	11.36/64.52	−53.16 (−59.01, −47.31)	<0.01	11.96/65.28	−53.32 (−57.89, −48.75)	<0.01
BEM vs IS	11.36/66.40	−55.04 (−60.87, −49.21)	<0.01	11.96/67.28	−55.32 (−59.85, −50.79)	<0.01
Otsu vs IS	64.52/66.40	−1.88 (−2.02, −1.74)	<0.01	65.28/67.28	−2.00 (−2.12, −1.88)	<0.01

Fifty oral biofilms were thresholded with four different methods. Half the biofilm images had been treated with water 8 and 18 hours into their 22 hour development and were designated as treatment biofilms. The other half were developed undisturbed over 22 hours and designated as control biofilms. The null hypothesis states that the mean difference between sets of thresholds obtained from one method vs another method is zero. Since all 12 null hypotheses were rejected, we conclude that each thresholding method was different from one another and is unaffected by treatment status of the images operated on. Although mean thresholds for Otsu and IS were roughly 2 intensity values apart, IS thresholds were consistently 2 units higher than Otsu thresholds applied to the same image, minimizing standard deviation and producing significant effects.

^a^For an image’s individual manual threshold value, the five values given by our five operators were averaged.

### Determination of biovolume, surface area, and number of objects following each thresholding method

The post-thresholding oral biofilm architectural measurements are summarized in Table 1 and visualized in Supplementary Fig. 1. Regardless of thresholding method, the biofilms treated with water at 8 and 18 hours had lower end-stage biovolume and surface area. In all four thresholding methods, biovolume and surface area differences between the two sets of biofilms were significantly different as tested by student’s 1-tailed heteroscedastic t-test with moderate effect sizes.

For objects detected, only the BEM method (p = 0.018) indicated significant differences between treatment and control biofilm images whereas IS (p = 0.101), Otsu (p = 0.114), and manual (p = 0.272) methods did not. The less aggressive BEM method detected decreased number of objects in the water-treated biofilms (2,521 objects) compared to control biofilms (3,526 objects). This difference in average number of objects detected was sufficient to produce a p-value of 0.018. Within the same sets of treatment and control images, Otsu (2,379 vs. 2,018), IS (2,433 vs. 2,051), and manual methods (1,244 vs. 1370) using higher thresholds detected no significant difference in the number of objects between treatment and control biofilms (Table 1).

In general, average biovolume and surface area from manual thresholding occupied a middle ground between BEM and IS/Otsu thresholds. An anomaly was the average number of objects detected using manual thresholds, which resulted in an average number of objects detected lower than all three automatic methods. For control and treatment images, manual thresholds detected an average of 1,370 and 1,244 objects, respectively. This indicates that many microcolonies of intensity 15–29 were filtered by the manual thresholds around the 30 region. However, as threshold is increased to the 60 region of Otsu and IS, existing large biomasses are fragmented into smaller ones, creating an influx of small objects (Supplementary Fig. 1). Out of the three automatic methods, BEM thresholds were closer to manual thresholding values.

### Visual comparison of the 22-hour growth oral biofilm before thresholding, after automatic thresholding, and after manual thresholding

**Figure 1:**
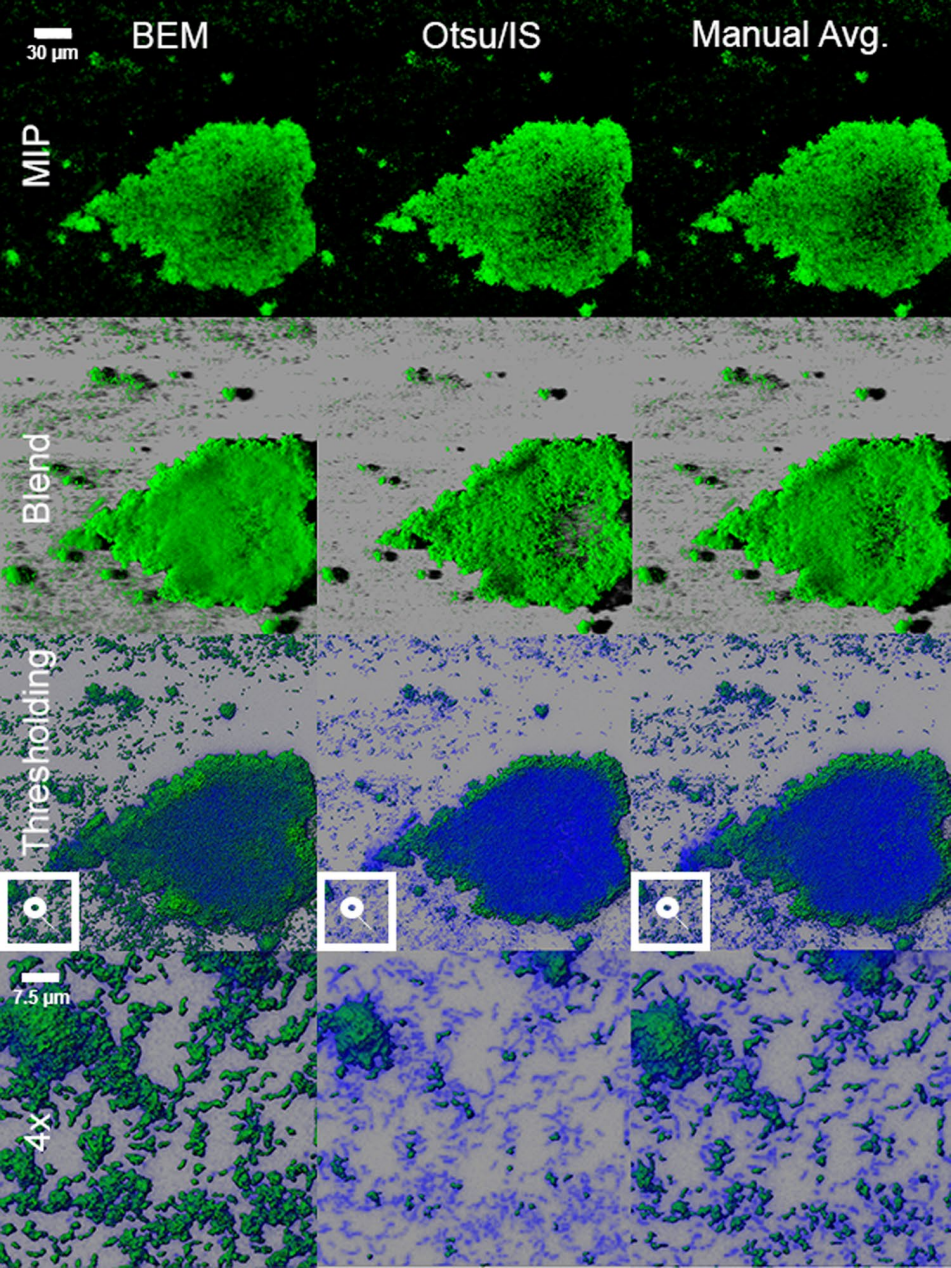
Visual Comparison of Different Thresholding Methods Applied to a Multi-Species Oral Biofilm. The maximum intensity projection, blend of all intensity values, and segmentation are shown from a top-down viewing angle of a CLSM image stack. The first, second, and third columns are the projection of the same confocal laser scanning microcopy image stack that is rendered after applying threshold selected by the BEM, Otsu/IS, and manual operators’ average, respectively. The MIP and blend projections show Otsu and IS methods threshold out the most biovolume, followed by manual and BEM methods. The “thresholding” projection in the third row shows biovolume that is above threshold in green, and biovolume that is below threshold in blue. The fourth row is a magnification from the lower left corner of the “thresholding” row and shows that Otsu and IS methods are too conservative in their thresholds. Low-intensity *Streptococcus* chains are lost after thresholding with Otsu and IS, leading to underestimates of actual biovolume. Manual threshold average is more comparable to BEM threshold in all three projection modes.

Figure 1 shows three rendering modes of the same image after application of BEM, Otsu/IS, and manual thresholds. All images were rendered with Imaris Software (Bitplane, Zurich, Switzerland). Otsu and IS post-threshold renders were virtually identical due to similar threshold values, and thus were grouped together in Fig. 1. Shadow projections of the same image with automatic thresholding and without thresholding are presented in the top half of Supplementary Fig. 1. After applying thresholds, the image was projected with maximum intensity projection (MIP), blend, and a hybrid mix of shadow projection and surface rendering. This hybrid projection is indicated by “thresholding” in row 3 of Fig. 1 and is designed to depict biomass that had been left intact and biomass that had been thresholded out. The BEM threshold of 10 differed visually from the Otsu and IS thresholds of 59 and 61, respectively (Fig. 1). The first row shows the MIP where each pixel’s given intensity value is the maximum out of all pixels at that location across the entire Z-axis. For the demonstration image, there are 53 slices. Thus, each pixel location has a set of 53 values. As compared to the other methods, The BEM MIP reveals more biofilm material in small flocs attached to the acquired pellicle. Additionally, the center of the large biofilm mass contains more signal (vis-à-vis biofilm) compared to the Otsu and IS thresholded images. The manual threshold average of 31.8 is intermediary to the results seen in BEM and Otsu/IS thresholds. The second row shows a blend render where the intensity of each pixel value, across the Z-axis, are blended together with inclusion of transparency. This mode allows for shadows within a 3D environment. In this mode, tiny flocs of biofilm material picked up by BEM’s lower threshold also are observed. Additionally, the cavitation in the middle of the biofilm is less pronounced.

The third row shows two superimposed renderings. The blue channel represents voxels that are removed by thresholding by each of the methods and the green channel represents voxels that are considered signal and true biovolume. The blue channel rendering has a transparency applied as to not mask the green signal render. Overall, the BEM-thresholded image captured the most biovolume, closely followed by manual threshold average. Additionally, the void space in the center of the large floc is less saturated with blue in the BEM image. The fourth row images are 4x snapshots of the high resolution render shown in the row above, where the focus is on the bottom left corner. At this magnification, it is evident that the blue haze that is removed by Otsu and IS thresholding is bacterial cells. There are numerous streptococcal signatures (multiple cocci cells arranged in chain-like morphology) that have been retained using the BEM method and lost with the other methods.

### Visual inspection of microspheres of known diameter after applying automatic thresholds

**Figure 2:**
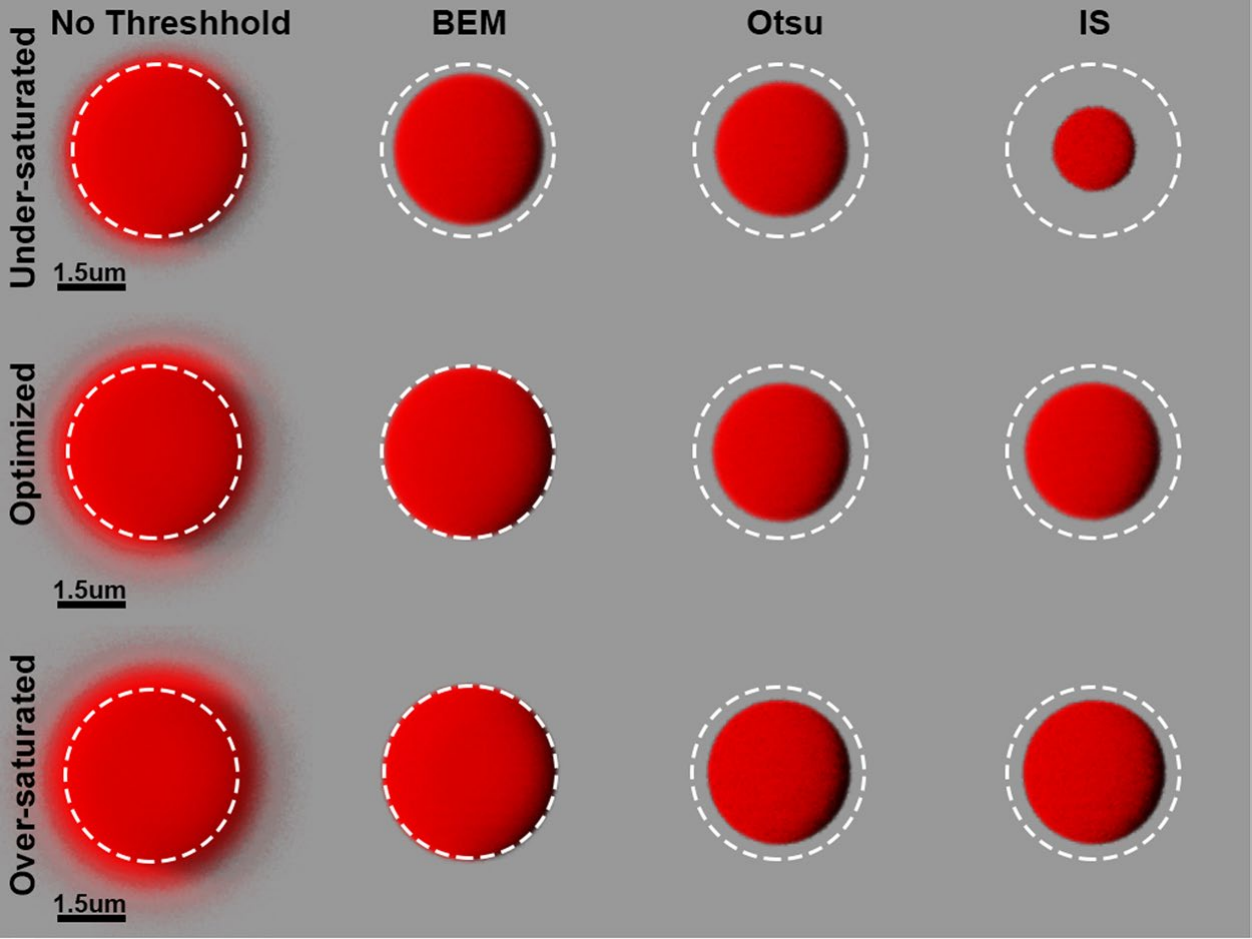
A Comparison of Microsphere Shadow Projection Renders Before and After Thresholding. Microsphere CLSM images with no threshold applied are characterized by a fuzzy halo surrounding the microsphere, indicating noise. After application of the BEM, Otsu, and IS thresholds, the fuzzy halo dissipates. The resultant microsphere object can then be compared by its diameter. The BEM threshold retains much of the microsphere signal, with its diameter close to four µm, the advertised diameter of the microsphere. Otsu and IS are too aggressive at removing signal belonging to the microsphere, resulting in diameters much smaller than four µm.

For validation of automatic thresholding techniques, the three automatic methods (BEM, Otsu, and IS) were applied to a fluorescent microsphere of known diameter and compared. Figure 2 shows post-threshold renders of a 4 µm diameter microsphere imaged with CLSM fluorescence. Three image stacks of the same microsphere were taken, each at a different gain: one stack each representing an under-saturated, gain-optimized, and over-saturated image. Thresholds calculated from the automatic methods of BEM, Otsu, and IS algorithms were applied to each image and rendered with Imaris using shadow projection mode (Table 3). Upon visual inspection, regardless of gain used in image capture, all three images showed a fuzzy halo around the microsphere when a threshold has not been applied. This was likely noise due to fluorescence of the object bleeding past its boundaries.

**Table 3:** Comparison of Calculated Diameter of Microsphere to its Expected Diameter.

Parameter	Under-saturated Microsphere One	Gain-Optimized Microsphere One	Over-saturated Microsphere One
BEM Threshold	13	11	11
Otsu Threshold	23	61	92
IS Threshold	59	63	94
X pixel length (µm)	0.048	0.048	0.048
Y pixel length (µm)	0.048	0.048	0.048
Expected Diameter (µm)	4	4	4
**Expected X pixel range (pixels)**	**83**	**83**	**83**
**Expected Y pixel range (pixels)**	**83**	**83**	**83**
No threshold X range (pixels)	113	140	157
No threshold Y range (pixels)	117	144	158
BEM threshold X range (pixels)	**74**	**81**	**85**
BEM threshold Y range (pixels)	**75**	**82**	**88**
Otsu threshold X range (pixels)	66	66	69
Otsu threshold Y range (pixels)	67	67	70
IS threshold X range (pixels)	41	66	69
IS threshold Y range (pixels)	43	67	69

One microsphere (Fig. 2 and Supplementary Fig. 2) was imaged with different gains to achieve under-saturated, gain-optimized, and over-saturated image histograms. Otsu and IS automatic thresholds calculated thresholds much higher than the BEM. The expected X and Y range for a single microsphere is 83 pixels. Images without thresholding result in a microsphere diameter that far exceeds the expected value. This excess diameter is noise due to the fluorescence of the microsphere object. BEM thresholding results in a calculated diameter closest to the expected value. In the gain-optimized and over-saturated images, diameter of the microsphere is very close to 4 µm. Otsu and IS thresholding results in a calculated diameter that is smaller than expected.

For each image, the BEM calculated the lowest thresholds, followed by Otsu’s method and IS (Supplementary Fig. 2). In the under and over saturated images and the gain-optimized image, the BEM values eliminated the fuzzy halo seen in the images without a threshold applied. The same observation can be made for Otsu and IS thresholds. Using the scale bars as a reference, the application of BEM thresholding resulted in a microsphere with a diameter that is closest to the expected value in all three images (Table 3). In the under-saturated image, the IS method calculated a high threshold value; this resulted in an underestimation of the diameter of the microsphere by roughly 50 percent. The application of Otsu’s method resulted in microspheres of comparable size in each image. Regardless of thresholding method, the diameter of the microsphere appears to increase as gain is increased.

### Comparison of automatic thresholding methods on an oral biofilm captured with different gains

**Figure 3:**
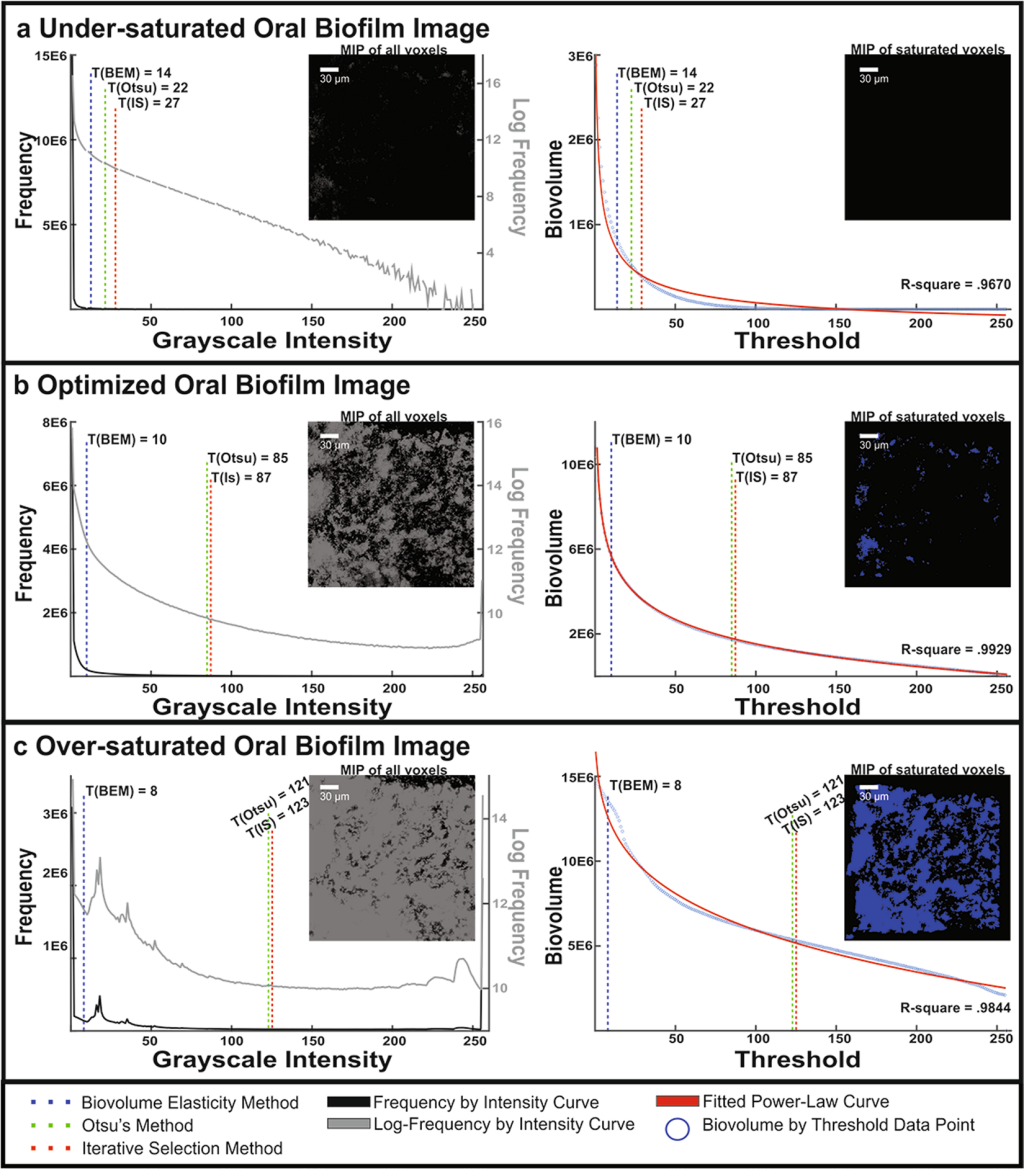
A Comparison of Image Histograms and Biovolume by Threshold Curves on an Oral Biofilm Taken with Three Different Gains. Three CLSM image stacks were acquired with identical image acquisition parameters except for gain. The first column shows grayscale histograms as well as the maximum intensity projection of the biofilm image shown in gray. The second column shows biovolume as a function of threshold, the fitted power law curve, and the maximum intensity projection of saturated voxels shown in blue. (**a**) Scenario where signal sensitivity is too low, yielding no saturated voxels. In this scenario, the BEM, Otsu, and IS are comparable in threshold detection. (**b**) Scenario where signal sensitivity is optimized by a confocal operator for the stain mixture, producing saturated voxels. BEM selects for a lower threshold compared to Otsu and IS methods. (**c**) Scenario where signal sensitivity is too high. BEM threshold selection is no longer applicable whereas Otsu and IS methods show robustness to operator error or inexperience. Correlation coefficients are high in all three scenarios, with the highest belonging to the image optimized by an operator.

Given that the diameter of the microsphere appears to increase as gain is increased, we explored further the effect of gain on the performance of the automatic thresholding methods, by imaging an oral biofilm with visually complex architecture using three different gains (Fig. 3). The under-saturated image stack was not sensitive enough to capture biofilm biomass that was clearly visually conspicuous and architecturally ornate when observed through the objective lens. Acquiring an image stack with insufficient gain resulted in a grayscale histogram that was severely weighted toward the low-intensity values (Fig. 3). In the portion of the histogram that was toward the higher intensity values, the log frequency values became erratic, indicating noise. For this scenario, where gain is insufficient and the stack is under-saturated, all three automatic thresholding methods were comparable. The BEM, Otsu, and IS methods calculated thresholds were 14, 22, and 27, respectively. The biovolume dropped precipitously as threshold increased due to low abundance of voxels containing signal. For this under-saturated stack, no voxels were saturated with high intensity (255) signal and the highest detected intensity was 230.

Using the Leica *Look Up Table* (LUT) interface, an optimal gain value was used to generate an image stack that contained the entire dynamic range of intensity values between 0 and 255. This “optimal” is achieved when biofilm starts showing saturated pixels (shown in blue within the LUT interface), indicating the entire dynamic range of 8-bit intensities are utilized. The image stack derived from using the optimal gain resulted in an image that was visually representative of the biofilm observed by eye through the objective lens. At this gain, the grayscale histogram was still weighted toward the low-intensity portion, but not as severely as the under-saturated image stack (Fig. 3a,b). The log frequency was more uniformly distributed across the intensity axis. In this gain-optimized image, the BEM-calculated threshold of 10 differed significantly from the thresholds calculated by Otsu (threshold of 85) and IS (threshold of 87). The maximum intensity projection of saturated voxels revealed a sporadic, but non-random presence along the X-Y Cartesian coordinate system. There were pockets of saturated voxels, indicating regions where biofilm had taken in the LIVE/DEAD^TM^ stain exceptionally well. It should be noted that the image histograms of all 50 oral biofilm images resembled the gain-optimized image histogram.

In the over-saturated image stack acquired with maximum gain, the biofilm fluorescent biovolume seemed greater than the biovolume, when viewed through the objective lens. The grayscale intensity histogram was similar in shape to the log-frequency histogram and both reveal a sharp peak at 0 and 255, but also a modality in the region of 20. At maximum gain, the BEM-calculated threshold of 8 remained disparate from Otsu and IS thresholds of 121 and 123, respectively. Additionally, the biovolume was least elastic to changes in threshold as compared to the two other tested gains. The MIP of saturated voxels revealed that over half the X-Y coordinates contained a saturated voxel.

The effect of gain on automatic thresholding methods were also evaluated using microspheres. A microsphere captured with three different gains was analyzed for its image histogram distributions and biovolume by threshold curves (Supplementary Fig. 2). Thresholds of microsphere images calculated by automatic methods followed the general observations seen in the oral biofilm images. BEM calculated the lowest thresholds, followed by Otsu’s method and IS, but BEM threshold ranges were tighter than Otsu and IS ranges.

### Quantitative assessment of automatic thresholding methods in boundary determination

The microsphere featured in Fig. 2 and Supplementary Fig. 2 was also used to assess the accuracy of post-thresholding measurements. With each X and Y pixel lengths known, the expected X and Y range of the largest object (i.e. the internal distance between the boundaries of the microsphere in the X and Y dimension) can be calculated. Under the imaging parameters used, the expected X and Y range is 83 pixels given the manufacturer’s claim that the microspheres are 4 µm in diameter. The thresholds applied to the under-saturated, gain-optimized, and over-saturated microsphere images and the calculated X and Y pixel ranges are shown in Table 3.

In the images that have not been thresholded, the X and Y run lengths of the largest object far exceed the expected range of 83 pixels. This is to be expected due to the noise seen in the no threshold renders of Fig. 2. As the gain is increased, the X/Y run lengths also increased from 113/117 pixels for the under-saturated microsphere to 140/144 pixels in the gain-optimized microsphere and from 140/144 pixels to 157/158 pixels in the over-saturated microsphere (Table 3). The thresholding method that achieved an X and Y run length closest to the expected range was the BEM. Otsu and IS were aggressive at removing signal, resulting in a microsphere of smaller dimensions than what was anticipated. The X and Y diameters calculated in Table 3 match the renders of the microspheres shown in Fig. 2.

### Quantitative assessment of automatic thresholding methods to remove background fluorescence

The same microsphere images used in Fig. 2 and Supplementary Fig. 2 were used to calculate total number of objects detected. Objects are distinguished from one another using a 26-neighborhood connectivity rule (Supplementary Fig. 4). The expected counts for the under-saturated, the gain-optimized, and the over-saturated images are all one. The additional objects detected can be considered background fluorescence due to the parameters of image acquisition. Post-thresholding removal of small objects are also assessed ranging from singleton voxels to quadrupleton voxels. The counted objects after applying each threshold and any post-thresholding processing are detailed in Table 4.

**Table 4:** Comparison of Calculated Objects Detected to its Expected Objects Detected.

	Parameter	Under-saturated Microsphere One	Gain-Optimized Microsphere One	Over-saturated Microsphere One
	Expected Number of Objects	1	1	1
No Threshold	All Objects Detected	1,103	3,521	10,799
	Objects Excluding Singletons	215	823	1,668
	Objects Excluding Doubletons	83	329	720
	Objects Excluding Tripletons	28	178	391
	Objects Excluding Quadrupletons	11	100	244
BEM	All Objects Detected	**1**	39	113
	Objects Excluding Singletons	**1**	**1**	13
	Objects Excluding Doubletons	**1**	**1**	**1**
	Objects Excluding Tripletons	**1**	**1**	**1**
	Objects Excluding Quadrupletons	**1**	**1**	**1**
Otsu	All Objects Detected	**1**	**1**	2
	Objects Excluding Singletons	**1**	**1**	**1**
	Objects Excluding Doubletons	**1**	**1**	**1**
	Objects Excluding Tripletons	**1**	**1**	**1**
	Objects Excluding Quadrupletons	**1**	**1**	**1**
IS	All Objects Detected	**1**	**1**	2
	Objects Excluding Singletons	**1**	**1**	**1**
	Objects Excluding Doubletons	**1**	**1**	**1**
	Objects Excluding Tripletons	**1**	**1**	**1**
	Objects Excluding Quadrupletons	**1**	**1**	**1**

One microsphere (Fig. 2 and Supplementary Fig. 2) was imaged with different gains to achieve under-saturated, gain-optimized, and over-saturated image histograms. Thus, expected number of objects detected is one. Background fluorescence can be detected in the number of objects detected beyond one. No thresholding results in the most number of objects detected. BEM thresholding, although sensitive, can be bettered by applying a post-thresholding processing step where singleton or doubleton voxels are eliminated. Otsu and IS, due to their aggressive and high thresholds, eliminate virtually all background fluorescence.

The three images with no threshold applied contained the most number of objects. This can be seen visually in Fig. 2. Filtering small objects ameliorated calculated outcomes, but still contained detectable background fluorescence even after object with volumes of up to four voxels are removed (Table 4). After application of the BEM threshold, the under-saturated image detected one object without need for further processing. In the gain-optimized image, 39 objects were detected after applying the BEM threshold compared to 3,521 objects detected with no threshold. This indicates 3,482 objects were signaled by low-intensity 8-bit values between one and eleven with eleven being the BEM threshold for the gain-optimized microsphere. After eliminating singleton voxels, only one large object remained, which represented the microsphere. Thus, 38 of the 39 objects were singleton voxels with an 8-bit value above 11 (all 38 singleton voxels had 8-bit intensity of 12 or 13). The spatial location of the 38 singleton objects were within one µm from the surface of the microsphere, suggesting the fluorescent noise is dependent on its proximity to the microsphere and is not uniformly distributed across all void space (data not shown). As the gain is increased, the BEM detected more objects, but such a situation is corrected by removing doubleton and singleton objects. The high thresholds calculated by Otsu and IS methods, in general, resulted in one object representing the microsphere. The instances where this is not observed was in the over-saturated image with two objects being detected (Table 4). The superfluous additional object was a singleton voxel.

### Effect of increasing percentage surface area coverage of fluorescent signal

The under-saturated, gain-optimized, and over-saturated microsphere images were each cropped from their original 512 × 512 pixel image down to 128 × 128 pixels. This process did not affect the image’s resolution. The X & Y pixel length remained the same. However, this process increased the percentage surface area coverage of the microsphere. It is suggested that for differential surface area coverage by fluorescent signal, the BEM functions the same whereas IS and Otsu calculates higher thresholds for images with higher percentage surface area coverage (Supplementary Table 1). As the Otsu and IS thresholds increase due to the increased field of view coverage, the calculated X and Y lengths also decreases, moving them further away from the expected range of 83 pixels.

Lastly, as surface area coverage by the microsphere increased, the number of objects detected decreased (Supplementary Table 2). This is expected as the field of view decreases, providing less of an opportunity for background fluorescence to be detected. The decreases in objects detected are more pronounced in the under-saturated, over-saturated, and gain-optimized images of Microsphere One with no threshold applied. This indicates the presence of background fluorescent signal far away from the microsphere. Roughly 8,000 objects in the 512 × 512 over-saturated image are lost when the image was cropped to a 128 × 128 window framing the microsphere, indicating these objects are on the periphery and not within proximity to the microsphere.

### Effect of increasing resolution of images containing fluorescent signal

Supplementary Tables 3 and 4 show the effects of increasing resolution on automatic thresholds, calculated microsphere diameters post-thresholding, and number of objects detected post-thresholding. As resolution increases, BEM, Otsu, and IS thresholds remain relatively inelastic. In the highest resolution image, BEM threshold increased by one and the Otsu and IS thresholds decreased by one. The BEM thresholds from the three images of varying resolutions calculated diameters that were extremely close or identical to the expected values. In all three images, Otsu and IS thresholds resulted in microspheres with diameters that underestimated the expected diameter.

Supplementary Table 4 shows that as resolution increases, so do the number of objects detected. In the 512 × 512 image thresholded using BEM, a filter for singletons was sufficient to reduce the number of objects down from 46 to one. Filtering singletons was not sufficient in the 1024 × 1024 image thresholded using BEM. Only when a filter that eliminates quadrupletons or below is applied do we see a single object in the 1024 × 1024 image thresholded using BEM.

### Z-axis effect on fluorescent signal

Supplementary Fig. 3 shows the distribution of signal intensity can vary by Z-slice. For the oral biofilm (Fig. 1) the middle slice is slightly more weighted toward the low-intensity spectrum. This is likely due to reaction and diffusion limitation due to thick biofilm that obstructs stain penetration^35^. This results in the cavitation seen in blend render mode of Fig. 1. Due to its small size, the fluorescent signal of the microsphere does not decay along the Z-axis as the stain is evenly distributed across the dimensions of the microsphere (Supplementary Fig. 3).

## Discussion

This work demonstrates the utility of a sensitive method to threshold image stacks of biofilms generated with a CLSM, which we called the biovolume elasticity method (BEM). BEM is a model-based approach that applies a criterion to the biovolume by threshold curve. Threshold is determined where biovolume becomes relatively inelastic to changes in threshold. To evaluate BEM, we compared it to two existing automatic methods – Otsu and iterative selection (IS), which rely on intensity histograms to calculate optimal thresholds. Otsu’s method is ubiquitous across many analytical packages and iterative selection had been shown in studies to be congruent with manually-set thresholds for biofilms captured with light microscopy and CLSM^13–15,30^. However, these methods are optimized for distinct intensity histograms that poorly fit the functional form of CLSM-acquired images of biofilms using fluorescence-based tagging^33,36^, resulting in high thresholds. At these elevated thresholds, our renderings showed that meaningful biofilm biomass is sequestered from the image stacks (Fig. 1), which could lead to biased analyses.

In 2001, Yang and colleagues compared five algorithms to threshold confocal biofilm images and concluded iterative selection (IS) was the most suitable method. The images used were of mono-species biofilms grown in a flow-cell reactor and captured with light microscopy and CLSM. They did not evaluate Otsu’s method. Their work, suggesting that IS functioned well enough to potentially replace manual operators in such images^30^, motivated our inclusion of IS for comparison with BEM and Otsu. However, we discovered that the IS method was very similar to Otsu’s method for all biofilm CLSM images. Yang and colleagues focused their panel of algorithms on light microscopy images, which are unimodal with the mode located at a middling intensity. However, for confocal images where the histogram is unimodal with a strong right-skew, Otsu’s method and IS are less easily differentiated.

Reliability between the five biofilm image analysis operators, who performed manual thresholding, was evaluated with intraclass correlation coefficients (ICC) calculated with both the consistency and agreement arguments as outlined by Koo *et al*. and Kim^37,38^. Consistency measures whether operators’ rank-order of ratings were similar whereas agreement measures whether the raters’ ratings are similar in magnitude. A 95% confidence interval was calculated for each ICC value to determine significance from a null hypothesis of ICC = 0. The intraclass correlation coefficients for absolute agreement and consistency amongst the five independent operators were 0.243 (−0.040 < ICC < 0.517) and 0.707 (0.556 < ICC < 0.818), respectively. This indicates that although the manual operators had preferences that lead to differences in magnitudes, their rank-order of thresholding by image was similar. The high variance of manually-calculated thresholds indicate that some individual operators prefer higher or lower thresholds than other operators (Table 1). However, within the set of images provided to them, they generally agree on which images should have the lowest thresholds and which images should have the highest thresholds. This highlights the concern that post-thresholding manual outcomes can vary depending on the nuances and preferences of an individual.

Manual thresholding can be compared to automatic methods, but perhaps should not be the gold standard for comparison of threshold methods. A standard could be post-threshold renders that provide the most biologically-sensible representation of biofilm. As described in Baveye’s comments to the 2001 study by Yang and colleagues, manual operators, when confronted with image histograms, tend to shy away from vigorous threshold values in favor of a compromise of what is intuitively deemed to be a reasonable balance between background and foreground^30,34^. This is functionally inherent in algorithms used in IS and Otsu’s methods and thus, the thresholds selected by manual operators using only image histograms may or may not be biologically relevant. Thresholds calculated using BEM were more inclusive of biologically relevant signal, such as streptococcal cells and visible micro colonies. Manual operators were less conservative than IS/Otsu, but not seemingly bold enough to place thresholds as low as BEM thresholds.

The low thresholds calculated by BEM were validated for fluorescent signal using fluorescent microspheres of known diameter. Two separate microspheres were imaged to test the rigor of BEM, Otsu, and IS methods under different gains and resolutions. Comparisons of post-threshold outcomes to expected outcomes were used to assess accuracy of automatic thresholding methods. Under all gains and resolutions tested, images treated with BEM thresholds resulted in microsphere diameters closest to 4 µm. The two-dimensional diameter was used instead of three-dimensional biovolume because Z-elongation artifacts on small objects make expected biovolume in voxels difficult to calculate^39^. Images treated with Otsu and IS thresholds resulted in microsphere diameters that were smaller than expected, suggesting that these two methods are too aggressive at removing meaningful fluorescent signal. Although the low BEM thresholds can result in more spurious background fluorescence being detected, this can be eliminated by applying a post-thresholding filter of singleton voxels in gain-optimized images.

The BEM is not without limitation. For example, using BEM to threshold confocal biofilm image stacks flooded with too much saturated voxels is not recommended. The BEM performs best when operating on a confocal image stack that has been gain-optimized with use of the entire dynamic range without saturation. In its current configuration, it is not recommended to apply BEM to 12-bit images, as the calculated thresholds remains around the 10 region out of a dynamic range of 4096. This essentially applies no threshold, leaving traces of background fluorescence (data not shown). This can be rectified by changing the percent change in slope criteria in order to scale the 8-bit thresholds to the dynamic range of a 12-bit image. For example, a threshold of 10/256 calculated for an 8-bit image would translate to a threshold of 160/4096 for an identical image in 12-bits. This underscores the importance of an adjustable criterion, such as a fixed percent change in slope. Otsu and IS performances can vary drastically between images of different bit-depths, but their performance cannot be adjusted due to their maximization criterions.

Our study highlights the importance of thresholding in defining biofilm properties. In particular, biofilm outcome measurements can be sensitive to thresholding methods. Automatic methods eliminate issues of operator subjectivity and inter-rater reliability that are inherent to manual methods, but are not a panacea to thresholding. Investigators must consider their image histograms and image-acquisition parameters prior to selecting an automatic method. For CLSM image stacks that produce unimodal histograms with an extended tail, the BEM is a sensitive alternative to IS/Otsu’s and manual methods. By calculating lower thresholds, the biovolume elasticity method minimizes data loss and retains low-intensity architecture.

## Methods

### Biovolume Elasticity Method (BEM)

The BEM is a model-based approach to global image segmentation whereby parameters to the model are optimized based on *a priori* expectation of oral biofilm shape and size^40^. The optimal BEM threshold is calculated by plotting the biovolume as a function of threshold. First, biovolume is calculated as the sum of all foreground voxels after a threshold is applied. Thus, the biovolume plots (right column Fig. 3) are directly related to the histograms (left column Fig. 3): biovolume is simply the sum of voxels with histogram values above the chosen threshold. However, plotting biovolume directly rather than using the histogram allows us to tune our approach based on the outcome measures we are aiming to estimate. The higher the threshold, the more stringent the criteria for a voxel to be classified as foreground, leading to decreased biovolume estimates.

Second, we plot biovolume across all 256 data points and fit a 2-term power curve which takes on the functional form of

1 $$bv(t)=a\,\ast \,{T}^{b}+c$$

where *bv* denotes biovolume, *T* denotes threshold, and *a*, *b*, *c* denote fitted constants. A power curve was chosen because we expected biovolume by threshold curve to possess a long tail^33^. Additionally, it provides excellent fits in the lower threshold region where our criterion is applied.

Third, the best-fit curve is differentiated to obtain the slope at each threshold, representing the sensitivity of biovolume estimation due to a unit change in threshold. Lastly, the criterion for selecting optimal threshold in our proposed biovolume elasticity method is the first instance where a one unit increase in threshold changes the slope by less than 10% as shown in Eq. 2:

2 $$\frac{bv^{\prime} (T+1)-bv^{\prime} (T)\,}{bv^{\prime} (T)} < 0.10$$

The 10% criterion is adjustable and was chosen for this study to select the most sensitive thresholds that can accurately define the boundaries of a microsphere of known diameter.

There are three assumptions of the BEM. The first is the image histogram of 8-bit grayscale values is unimodal with an extended tail (Fig. 2a). Second, the mode is located at intensity value of 0. Lastly, the entire dynamic range (0–255) is utilized without saturation of 255 voxels (Fig. 3b). The change in slope criterion can be tuned to scale for higher bit images.

### Otsu’s method

The threshold from Otsu’s method was calculated using MatLab’s graythresh function. Otsu’s method seeks to maximize interclass variance, or equivalently, minimize intraclass variance. The formulation is detailed in the original proposal of the method^28^. Otsu’s method has two main assumptions. The first is that the image histogram is bimodal, indicating separation of grayscale intensities of foreground object and background. The second is that there is a clear sharp valley between the two modes. As the size of the peaks become more disparate in size to each other, or if the image is corrupted by noise, the identifiable valley becomes less transparent, and Otsu’s method is more prone to error.

### Iterative Selection method (IS)

The threshold from the IS method was calculated using a MatLab script coded to the specifications described by Yang *et al*.^30^. Briefly, IS places the threshold where the difference between mean intensity values of background pixels and mean intensity values of foreground pixels is maximized. This functionally provides the most contrast between objects and background. The IS has two assumptions. The first is that the image histogram is bimodal. The second is that the image possesses objects that have average mean intensity that is distinguishable from the average mean intensity of the background^29^.

### Manual thresholding

Manual thresholding was also performed on each of the 50 acquired images. Five operators with experience rendering biofilm images with Imaris (Bitplane, Zurich, Switzerland) manually thresholded each image. The manual operators were presented the grayscale intensity histograms of each image to manually threshold and were instructed to select a threshold value where the foreground intensity signal is linearly distributed. Operators were blinded to treatment status. The five manual thresholds for each image were averaged, rounded to the nearest integer, and used for biovolume, surface area, and object detection.

### Post-thresholding filtering of small objects

After a threshold was applied, the binary image was further processed. Using MatLab’s bwareaopen function, singleton voxels were removed to eliminate background fluorescence.

### Development of oral multi-species biofilms for testing automatic and manual thresholding methods

Ten oral biofilms were developed overnight using 24-well microfluidic plates on the Bioflux 200 (Fluxion Biosciences, San Francisco, CA). The 24-well system is an adaptation of the 48-well Bioflux^TM^ system that has demonstrated reproducibility in developing biofilms representative of early supragingival plaque^41,42^. The 24-well system features a secondary inlet well which enables the introduction of a treatment regimen concomitantly with media infusion. We used the media and inoculum collection protocol described by Samarian *et al*.^42^ that uses pooled saliva for the inoculum (cell-containing saliva, CCS) and pooled cell-free saliva for the media (CFS). The saliva collection protocol was reviewed by the University of Michigan Institutional Review Board for Human Subject Research and deemed “not regulated”.

To inoculate a 24-well plate, cell-free saliva media (CFS) was flowed backwards from outlet wells to inlet wells to coat the viewing port. The CFS was incubated at room temperature for 20 minutes to allow for acquired pellicle formation. Cell-containing saliva inoculum (CCS) was then added from outlet wells to inlet wells and incubated for 1 hour at 37 °C to enable cells to adhere to the acquired pellicle. After incubation, the primary inlet wells were filled with 2 mL of CCS media and the secondary inlet wells were filled with 2 mL of sterile water. An automated protocol was set up with the Bioflux^TM^ software to supply the viewing port with media at a constant flow rate of 0.4 dyne/cm^2^. Five samples were grown uninterrupted for 22 hours while another five samples were treated with water at 8 and 18 hours into its 22 hour growth phase. Each treatment regimen was at 2.0 dyne/cm^2^ for two minutes.

At the end of the 22-hour growth, the remaining CCS media from the primary inlet well were aspirated and replaced with 1 mL of 1x PBS. The biofilm was washed at 0.4 dynes/cm^2^ for 20 minutes. After washing, the remaining PBS in the inlet wells were aspirated and the biofilm was stained with 3.34 µM Syto-9 and 20 µM propidium iodide solution at 0.4 dynes/cm^2^ for 40 minutes. The stained biofilms were subsequently washed by flowing PBS through the system at 0.4 dynes/cm^2^ for 20 minutes.

### Image acquisition of oral multi-species biofilms

A Leica Model TCS SPE (Leica Microsystems, Buffalo Grove, IL) inverted confocal laser scanning microscope equipped with an air immersion objective lens (NA 0.85, 40x magnification, model HCX PL APO) was used to capture biofilm stacks from the Bioflux^TM^ viewing ports. Excitation of stain mixture was achieved with a 488 nm solid state laser. Emission capture parameters were standardized for the stain concentration used in the experiment and unchanged between plates. Specifically, these parameters included: 15% laser intensity of the 488 nm laser, 900 V gain, −7.6% smart offset, and 1.00x digital zoom. Each biofilm channel was imaged five times at five locations along the viewing port using the same image acquisition parameters. To provide an objective methodology of imaging a heterogeneous biofilm, five locations of the viewing port imaged were determined *a priori* and correspond to roughly the beginning of the viewing port, 1^st^ quarter, middle, 3^rd^ quarter, and the end of the viewing port. Gains of 726V, 900V, and 1250V were applied to one confocal stack to determine the effects of gain increases on thresholding performances. Additionally, an empty channel was stained with Syto-9/propidium iodide mixture and imaged at various depths to evaluate background noise distribution.

### Fluorescent microspheres of known diameter for testing automatic thresholding methods

A TetraSpeck^TM^ fluorescent microspheres slide embedded with 4 µm microspheres was used as a positive control to evaluate automatic thresholding methods. Two individual microspheres (referred to as Microsphere One and Microsphere Two in tables) were imaged with a Leica Model TCS SP5 equipped with a glycerol immersion objective lens (NA 1.30, 63x magnification, model HCX PL APO CS). Excitation of microspheres was achieved with a 561 nm laser at 20% intensity, 0.0% offset, and 10.0x digital zoom. Varying gains and resolutions were used to test effects of these imaging parameters on automatic thresholding algorithms.

Microsphere One was imaged at 512 × 512 resolution using 430V, 494V, and 540V gains to represent an under-saturated, optimized, and over-saturated LUT. The three images of Microsphere One were cropped to 128 × 128 pixels to increase the percentage surface area coverage of the microsphere (Supplementary Tables 1 and 2). Microsphere Two was imaged at a gain-optimized 496V at 256 × 256, 512 × 512, and 1024 × 1024 resolutions (Supplementary Tables 3 and 4). Diameters in pixels were calculated by taking the range of the X and Y coordinates from the largest object.

### Converting confocal stacks to MatLab readable format

Archives containing the confocal images were converted to the MatLab.mat format using the MatLab Exporter plugin in Icy^14^. Each image is represented by a X * Y * Z * T * C cell array in uint8 format where X,Y and Z represent a voxel in three-dimensional space, T represents a time point, and C represents channel. Contained within each cell is an 8-bit unsigned integer (0–255) corresponding to signal strength from image acquisition. Under the parameters of image acquisition, the only dimension that is variable is Z, which the confocal operator sets for each image depending on biofilm thickness at the location of image acquisition. The dimensions of X, Y, T, and C were constrained to 512, 512, 1, and 2 respectively. Since the biofilms used were highly viable and all images were endpoint acquisitions, the red channel and time dimension were disregarded, leaving a three-dimensional cellular array for each image. Automatic and manual thresholds were then applied to cell array data using Matlab 8.5.

### Post-thresholding calculation of core biofilm architectural outcomes

Once a threshold is determined manually or automatically with BEM/Otsu/IS, voxels with signal below or equal to the threshold are converted to background and voxels with signal above the threshold are retained as foreground. The core architectural outcomes calculated post-thresholding were biovolume, and surface area, and the number of objects detected. Object detection was done using the MatLab bwconncomp function using a 26-connectivity neighborhood criteria (i.e. so that two voxels count as ‘connected’ if they touch at any face, edge, or corner). An illustration of this type of object connectivity is shown in Supplementary Fig. 4 where two voxels with different connectivity are considered to be individual objects and the total objects detected would be three. The total number of detected objects is the sum of all objects detached from other signal via 26-connectivity rule. Biovolume is calculated as the sum of all foreground voxels at a threshold. Similarly, surface area is calculated as the sum of exposed surfaces of all foreground voxels at a given threshold. All three outcomes are sensitive to thresholds.

### Comparing thresholds placed manually and thresholds calculated automatically with BEM, Otsu, and IS

To compare magnitude differences between thresholding methods, a student’s paired t-test was used to evaluate whether mean threshold difference of one method versus another method was zero. This analysis was performed for each pairwise set of thresholding methods and was stratified by treatment and control images.

### Comparing biofilm architecture outcomes between treatment and control groups after four different thresholds are applied

Means of architectural outcomes post-thresholding between biofilms intermittently treated with water over 22 hours and biofilms grown over 22 hours were compared with Student’s 1-tailed t-tests. The treatment and control groups had sufficient sample size with 25 images each. We hypothesized that biofilms treated intermittently with water will have lower biovolume, surface area and total number of objects detected. Since biofilm architecture is intrinsically heterogeneous and as a consequence of our pre-established positioning for biofilm imaging, we expected the treatment and control measurements to come from distributions with unequal variances. Thus a more conservative heteroscedastic assumption was made and applied to the t-tests. A two-tailed t-test was used to compare average thresholds between treatment and control groups since we believed there to be no differences in thresholds between the groups. Significance threshold was set at α = 0.05.

## Electronic supplementary material

Supplementary Figures and Tables
