# Supplementary material for: A Sensitive Thresholding Method for Confocal Laser Scanning Microscope Image Stacks of Microbial Biofilms
Source: Sci Rep. 2018 Aug 29;8:13013. doi: 10.1038/s41598-018-31012-5 (PMC6115396; doi:10.1038/s41598-018-31012-5)
Supplement: Supplementary file 1 — Supplementary Figures and Tables [file 41598_2018_31012_MOESM1_ESM.pdf]

Supplementary Information

A Sensitive Thresholding Method for Confocal Laser Scanning Microscope Image Stacks of Microbial Biofilms

Ting L. Luo[1], Marisa Eisenberg[1], Michael Hayashi[1], Carlos Gonzalez-Cabezas[2], Betsy Foxman[1],

Carl F. Marrs[1], Alexander H. Rickard[1*]

[1] Department of Epidemiology, University of Michigan School of Public Health, Ann Arbor, MI, USA

[2] Department of Cariology, Restorative Sciences and Endodontics, University of Michigan School of Dentistry, Ann Arbor, MI, USA.

Supplementary Figures

Supplementary Figure 1. Post-thresholding shadow projection renders of two oral biofilm images. Thresholding is necessary to remove background noise of biofilm images captured with CLSM**.** The figure shows two sample CLSM image stacks. One image of a control and one image of a treatment oral biofilm. The views shown are top-down, angled, and side perspective views. **A)** Image that is not thresholded is visually compared to **B)** image that is thresholded with the biovolume elasticity method (BEM), **C)** Otsu's Method, and **D)** iterative selection (IS). Images that had been thresholded with the BEM display greater visual detail of biofilm structure. Background noise that decreases image sharpness is evident in images that have not been thresholded. Otsu and IS thresholding removes a significant amount of biomass. This is evidenced by the cavitation in the large biomass within the control oral biofilm that is seen after applying Otsu and IS thresholds. In the treatment image, thresholding with Otsu and IS eliminates streptococcal signatures, microcolonies, and reduces existing biomasses into smaller biomasses.

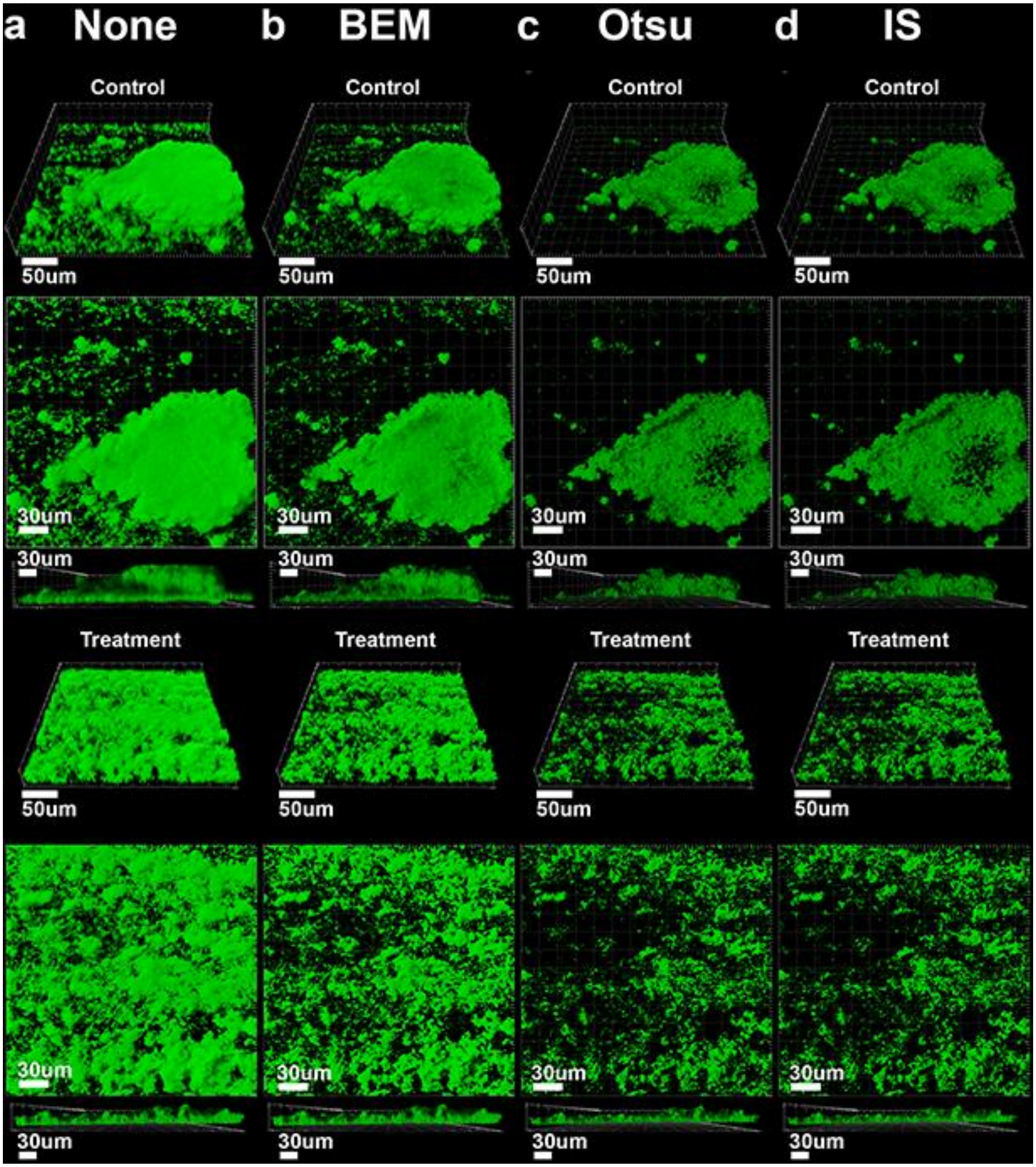
a None
b BEM
c Otsu
d IS
Control
Treatment
50um
30um

Supplementary Figure 2. A Comparison of Image Histograms and Biovolume by Threshold Curves of a Microsphere Taken with Three Different Gains. Three CLSM images of one microsphere were acquired with identical image acquisition parameters except for gain. The first column shows grayscale histograms as well as the maximum intensity projection of the microsphere image. The second column shows biovolume as a function of threshold, the fitted power law curve, and the maximum intensity projection of saturated voxels (shown in blue). **A)** In this scenario where signal sensitivity is too low, no saturated voxels were detected. In this scenario, BEM calculated the lowest thresholds, followed by Otsu and IS. IS threshold is noticeably larger than both other methods. **B)** In the scenario where signal sensitivity is optimized by a confocal operator to utilize the entire dynamic range (0-255). BEM selects for a lower threshold compared to Otsu and IS methods. **C)** In the scenario where signal sensitivity is too high, BEM threshold did not change whereas Otsu and IS methods show increased thresholds. In all three scenarios, correlation coefficients are high with the highest belonging to the gain-optimized image.

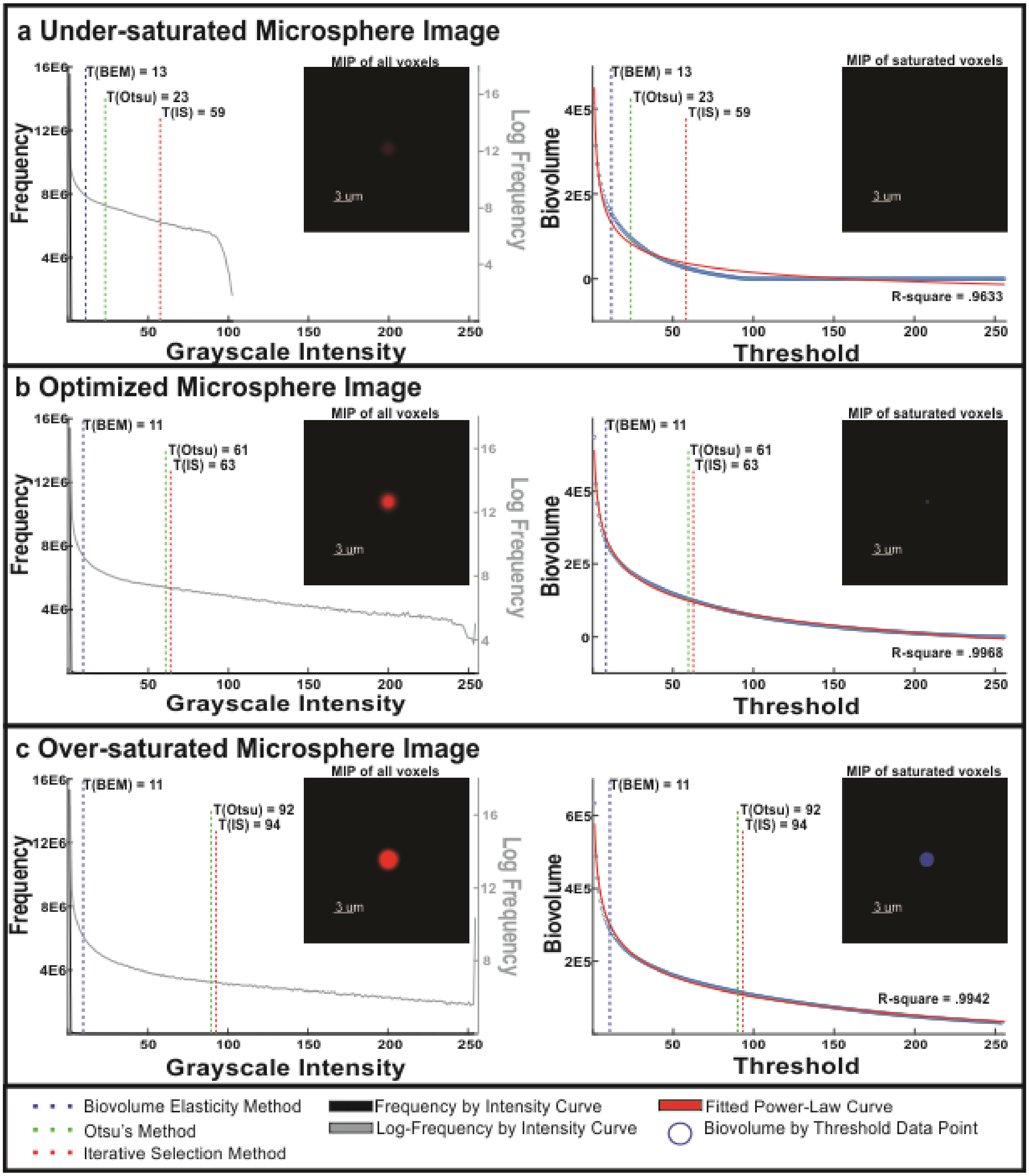

a Under-saturated Microsphere Image
T(BEM) = 13
T(Otsu) = 23
T(IS) = 59
MIP of all voxels
3 um
Frequency
Log Frequency
Grayscale Intensity
Biovolume
Threshold
MIP of saturated voxels
R-square = .9633
b Optimized Microsphere Image
T(BEM) = 11
T(Otsu) = 61
T(IS) = 63
R-square = .9968
c Over-saturated Microsphere Image
T(BEM) = 11
T(Otsu) = 92
T(IS) = 94
R-square = .9942
Biovolume Elasticity Method
Otsu's Method
Iterative Selection Method
Frequency by Intensity Curve
Log-Frequency by Intensity Curve
Fitted Power-Law Curve
Biovolume by Threshold Data Point

Supplementary Figure 3. Effect of Z-axis on fluorescent signal. The signal intensity histogram is taken from three slices each of the fluorescent microsphere (Figure 2) and an oral biofilm (Figure 1). For fluorescent microspheres, signal distribution is unaffected by Z-axis. In the oral biofilm image, fluorescent signal decays the closer the Z-plane approaches the middle of the biofilm. This indicates diffusion limitation of stain caused by thickness of the biofilm.

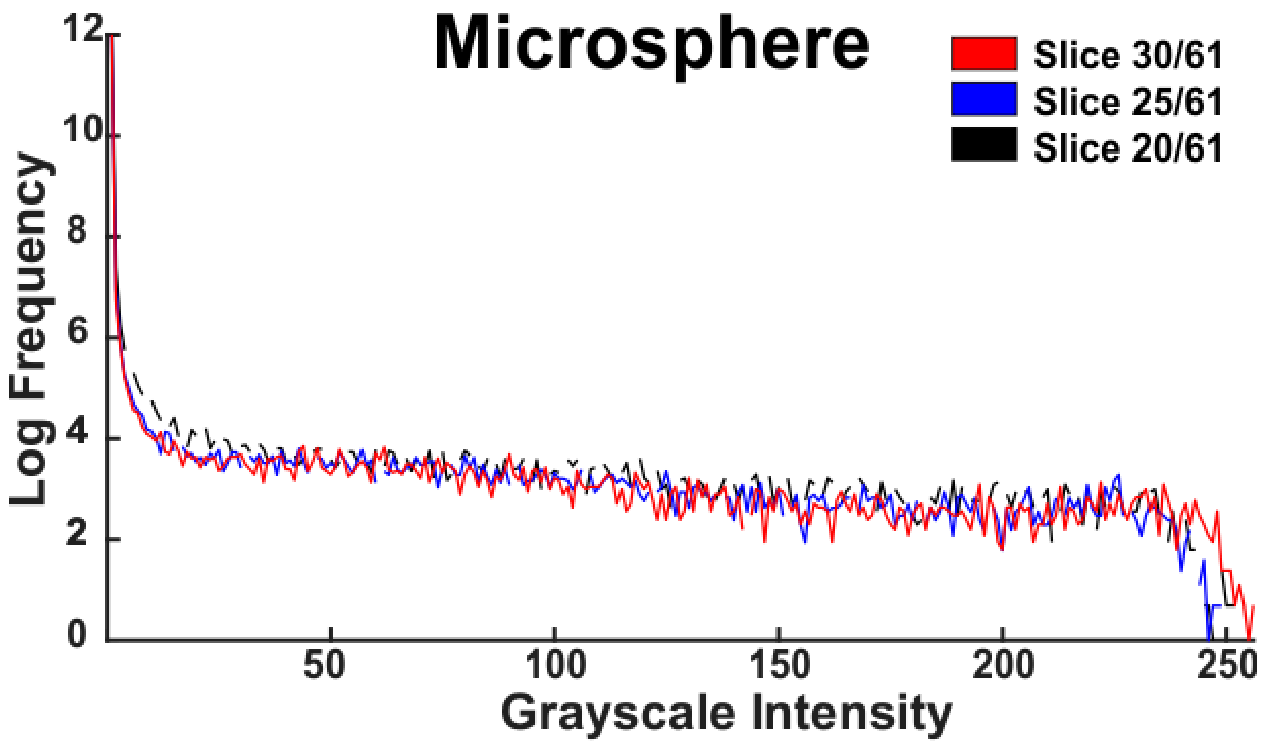
Microsphere
Slice 30/61
Slice 25/61
Slice 20/61
Log Frequency
Grayscale Intensity
12
10
8
6
4
2
0
50
100
150
200
250

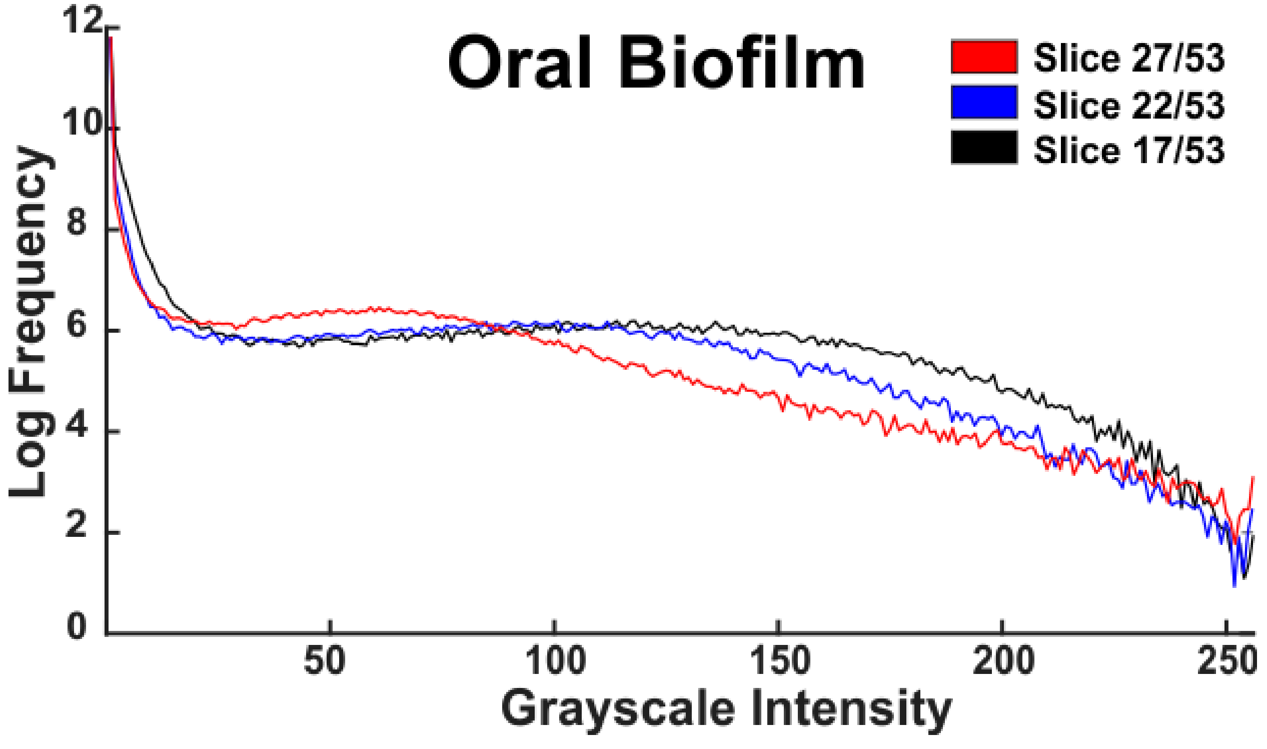
Oral Biofilm
Slice 27/53
Slice 22/53
Slice 17/53
Log Frequency
Grayscale Intensity
12
10
8
6
4
2
0
50
100
150
200
250

Supplementary Figure 4. Visual representation of the 26-neighborhood connectivity criteria. All three objects below are considered separate objects with a biovolume occupying 2 voxels. The object on the left has two voxels on the same plane connected by a plane at an interface. The middle object has two voxels on the same plane connected by a line. The object on the right has two voxels on different planes connected by a point.

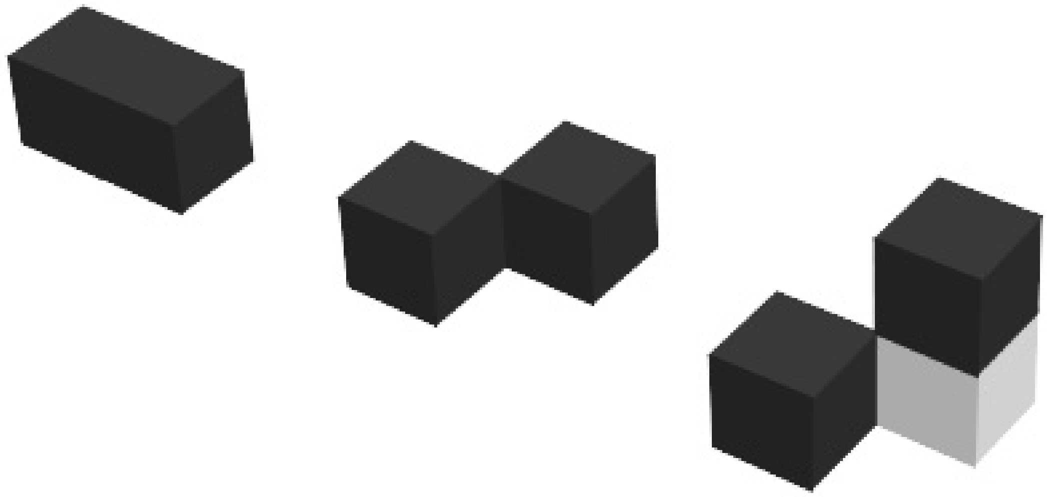

Supplementary Table 1. Testing effect of increased surface area coverage on automatic thresholds and calculated diameters. Values for the 512x512 image stacks are identical to Table 3. BEM threshold values are not affected by cropping images to achieve a higher percentage surface area coverage by the microsphere. Otsu and IS thresholds are mildly affected, increasing thresholds in the images with the higher surface area coverage by the microsphere. The calculated X & Y ranges slightly decrease for Otsu and IS, reflecting the increase in thresholds. Calculated X & Y ranges after applying BEM threshold remained unchanged. BEM ranges are closest to the expected ranges.

**Parameter**	**512x512/128x128 Under-saturated Microsphere One**	**512x512/128x128 Optimized Microsphere One**	**512x512/128x128 Over-saturated Microsphere One**
BEM Threshold	13/13	11/11	11/11
Otsu Threshold	23/25	61/66	92/98
IS Threshold	59/65	63/69	94/101
X pixel length(µm)	0.048/0.048	0.048/0.048	0.048/0.048
Y pixel length(µm)	0.048/0.048	0.048/0.048	0.048/0.048
Expected Diameter (µm)	4/4	4/4	4/4
**Expected X pixel range (pixels)**	**83/83**	**83/83**	**83/83**
**Expected Y pixel range (pixels)**	**83/83**	**83/83**	**83/83**
No threshold X range (pixels)	113/113	140/128*	157/128*
No threshold Y range (pixels)	117/117	144/128*	158/128*
BEM threshold X range (pixels)	**74/74**	**81/81**	**85/85**
BEM threshold Y range (pixels)	**75/75**	**82/82**	**88/88**
Otsu threshold X range (pixels)	66/64	66/65	69/68
Otsu threshold Y range (pixels)	67/65	67/66	70/68
IS threshold X range (pixels)	41/39	66/63	69/67
IS threshold Y range (pixels)	43/39	67/64	69/68

* Second value is restricted by the dimensions of the 128x128 image

Supplementary Table 2. Testing effect of increased surface area coverage on calculated objects. Values for the 512x512 image stacks are identical to Table 4. Increasing the surface area coverage by the microsphere, by cropping, decreases number of objects detected. The effects are more pronounced in the three images without a threshold applied. This indicates the presence of fluorescent noise far away from the microsphere. Roughly 8,000 objects in the 512x512 over-saturated image are lost when the image was cropped to a 128x128 window framing the microsphere.

	**Parameter**	**512x512/128x128 Under-saturated Microsphere One**	**512x512/128x128 Optimized Microsphere One**	**512x512/128x128 Over-saturated Microsphere One**
	**Expected Number of Objects**	**1**	**1**	**1**
**No Threshold**	All Objects Detected	1,103/1,089	3,521/2,508	10,799/2,638
	Objects Excluding Singletons	215/213	823/662	1,668/792
	Objects Excluding Doubletons	83/81	329/278	720/346
	Objects Excluding Tripletons	28/27	178/152	391/178
	Objects Excluding Quadrupletons	11/11	100/79	244/109
**BEM**	All Objects Detected	**1/1**	39/39	113/113
	Objects Excluding Singletons	**1/1**	**1/1**	13/13
	Objects Excluding Doubletons	**1/1**	**1/1**	**1/1**
	Objects Excluding Tripletons	**1/1**	**1/1**	**1/1**
	Objects Excluding Quadrupletons	**1/1**	**1/1**	**1/1**
**Otsu**	All Objects Detected	**1/1**	**1/1**	2/2
	Objects Excluding Singletons	**1/1**	**1/1**	**1/1**
	Objects Excluding Doubletons	**1/1**	**1/1**	**1/1**
	Objects Excluding Tripletons	**1/1**	**1/1**	**1/1**
	Objects Excluding Quadrupletons	**1/1**	**1/1**	**1/1**
**IS**	All Objects Detected	**1/1**	**1/1**	2/1
	Objects Excluding Singletons	**1/1**	**1/1**	**1/1**
	Objects Excluding Doubletons	**1/1**	**1/1**	**1/1**
	Objects Excluding Tripletons	**1/1**	**1/1**	**1/1**
	Objects Excluding Quadrupletons	**1/1**	**1/1**	**1/1**

Supplementary Table 3. Testing effect of increased resolution on automatic thresholds and calculated diameters. A microsphere was imaged three times using gain-optimized 496V. Technical replicates were achieved by imaging the same microsphere under different resolutions. The resolution of image stacks had minimal effect on threshold values. At the highest resolution of 1024x1024, BEM threshold increased by one and both Otsu and IS thresholds decreased by one. The BEM thresholds from all three images calculated diameters that were extremely close or identical to the expected values. In all three images, Otsu and IS thresholds resulted in microspheres with diameters that underestimate the expected diameter.

**Parameter**	**256x256 Resolution Microsphere Two**	**512x512 Resolution Microsphere Two**	**1024x1024 Resolution Microsphere Two**
BEM Threshold	10	10	11
Otsu Threshold	62	62	61
IS Threshold	64	64	63
X pixel length(µm)	0.096	0.048	0.024
Y pixel length(µm)	0.096	0.048	0.024
Expected Diameter (µm)	4	4	4
**Expected X pixel range (pixels)**	**42**	**83**	**166**
**Expected Y pixel range (pixels)**	**42**	**83**	**166**
No threshold X range (pixels)	70	144	311
No threshold Y range (pixels)	71	146	299
BEM threshold X range (pixels)	**41**	**83**	**164**
BEM threshold Y range (pixels)	**41**	**83**	**166**
Otsu threshold X range (pixels)	33	67	135
Otsu threshold Y range (pixels)	33	67	135
IS threshold X range (pixels)	33	67	134
IS threshold Y range (pixels)	33	66	135

Supplementary Table 4. Testing effect of increased resolution on calculated objects. A microsphere was imaged three times using gain-optimized 496V. Technical replicates were achieved by imaging the same microsphere under different resolutions. As resolution increases, the number of objects detected increases. This is particularly noticeable in the images that have no threshold applied. In the 512x512 resolution image, the BEM performed best when singleton voxels are filtered, yielding the expected value of 1. However, in the 1024x1024 resolution image, BEM needed a higher object size filter to detect only the microsphere object.

	**Parameter**	**256x256 Resolution Microsphere Two**	**512x512 Resolution Microsphere Two**	**1024x1024 Resolution Microsphere Two**
	**Expected Number of Objects**	**1**	**1**	**1**
**No Threshold**	All Objects Detected	249	3,040	47,832
	Objects Excluding Singletons	32	708	7,071
	Objects Excluding Doubletons	10	280	3,212
	Objects Excluding Tripletons	5	139	1,837
	Objects Excluding Quadrupletons	3	75	1,201
**BEM**	All Objects Detected	**1**	46	632
	Objects Excluding Singletons	**1**	**1**	94
	Objects Excluding Doubletons	**1**	**1**	24
	Objects Excluding Tripletons	**1**	**1**	5
	Objects Excluding Quadrupletons	**1**	**1**	**1**
**Otsu**	All Objects Detected	**1**	2	126
	Objects Excluding Singletons	**1**	**1**	5
	Objects Excluding Doubletons	**1**	**1**	2
	Objects Excluding Tripletons	**1**	**1**	**1**
	Objects Excluding Quadrupletons	**1**	**1**	**1**
**IS**	All Objects Detected	**1**	2	112
	Objects Excluding Singletons	**1**	**1**	4
	Objects Excluding Doubletons	**1**	**1**	2
	Objects Excluding Tripletons	**1**	**1**	**1**
	Objects Excluding Quadrupletons	**1**	**1**	**1**
